# Nutrition Considerations for Burn Patients: Optimizing Recovery and Healing

**DOI:** 10.3390/ebj4040035

**Published:** 2023-10-13

**Authors:** Beth A. Shields, Asia M. Nakakura

**Affiliations:** US Army Institute of Surgical Research, JBSA, Fort Sam Houston, TX 78234, USA; asia.m.nakakura.mil@health.mil

**Keywords:** nutrition, burn, nutrition support

## Abstract

The hypermetabolic and hypercatabolic responses to severe burns put nutrition support at the forefront of treatments. When left untreated, severe weight loss, increased infection, and wound healing failure can occur. Enteral nutrition is the primary method of nutrition support in such patients. Meeting caloric needs and a positive nitrogen balance are short-term goals of nutrition support, with long-term goals of minimizing lean body mass loss and maximizing wound healing. High-carbohydrate and low-fat nutrition received evidence from randomized controlled trials of aiding in decreasing pneumonia rates and was found to promote positive nitrogen balance, which lipids do not do. We go through the macronutrient and micronutrient needs of the burn patient as well as techniques for meeting these needs in the modern intensive care unit, with some discussion of alterations in these techniques that are required in the austere environment.

## 1. Introduction

Thermal injuries are a significant issue in both peacetime and wartime. Severe burns cause extensive physical trauma with a resulting cascade of metabolic alterations, including hypermetabolism and hypercatabolism. During the hypermetabolic response, the basal metabolic rate of a burn patient may be twice their normal rate, resulting in severe weight and lean mass loss and placing the burn patient at an increased risk for infection and delayed wound healing. These processes are the most extreme after severe burns, compared to other traumas and medical conditions, in addition to being much more prolonged [1]. Adequate nutrition is crucial for burn patients, to support the healing process and prevent complications. Austere wartime environments can pose significant obstacles to providing adequate nutrition. In these settings, individuals may face limited access to conventional hospital supplies, making it crucial to explore alternative strategies to ensure proper nourishment to promote wound healing. This manuscript aims to provide a review of the metabolic response to burn injury, discuss the nutritional assessment methods, explore the macronutrient and micronutrient requirements, and provide an overview of the nutrition support strategies in both peacetime and austere environments.

## 2. Nutritional Assessment in Burn Patients

The nutrition assessment of a burn patient includes obtaining pre-injury weight, height, medical history, biochemical data, medications, and physical examination data. A visible assessment prior to fluid resuscitation can be used to determine if temporal wasting or other signs of malnutrition are present. Recent weight loss and a timeline for any poor oral intake prior to injury should be determined when the patient can answer questions or if family members are present to provide this information [2]. The usual dry weight must be determined in order to properly use assessment calculations, as patients may be extremely hypervolemic (~25 kg of edema is common). The usual dry weight can be determined using the medical record, admission weight minus any intravenous fluids provided prior to admission if it is a recent burn, an identification card (driver’s license/identification card), or a report per the patient or family. Note that the weight cannot usually be used for nutritional monitoring until all edemas are resolved.

The increase in the resting energy expenditure is due to the systemic inflammatory response, with increased catecholamines and other acute phase reactants. Meeting the increased energy demands with the appropriate macronutrient intake is essential for promoting wound healing, preventing infection, and maintaining lean body mass. An accurate estimation of energy needs is crucial for avoiding underfeeding or overfeeding. Only one RCT has evaluated calorie goals for burn patients [3]. This RCT did not find any significant outcome differences between providing approximately 20% versus 40% above the resting energy expenditure. Unfortunately, there was a baseline difference in age between the higher and lower calorie groups (37 ± 9 vs. 29 ± 10 years; *p* < 0.05), which skewed the results in favor of the lower calorie group [3]. We previously found the Milner equation [1] to be the most satisfactory in determining the resting energy expenditure for patients with a ~ ≥20% TBSA burn when compared with indirect calorimetry [4]. The Milner equation is as follows:

Kcal/day = [BMR × (0.274 + 0.0079 × TBSA - 0.004 × PBD) + BMR] × 24 × BSA × AF

TBSA = total body surface area burned (%) × 100, ex.: 20% burn, enter “20”

Note: TBSA does not change with healing; always use the initial burn size

BSA (m^2^) (usual answers: 1.5–2.5) = body surface area

Square root of (HT × WT)/3600

HT = height (cm)

WT = weight (kg) (for obese pts, use actual dry weight)

AF = activity factor (typically 1.4 for weight maintenance [5,6,7] and 1 when paralyzed)

PBD = post-burn day

BMR = basal metabolic rate

Male BMR = 54.337821 − (1.19961 × Age) + (0.02548 × Age^2^) − (0.00018 × Age^3^)

Female BMR = 54.7494 − (1.54884 × Age) + (0.03580 × Age^2^) − (0.00026 × Age^3^)

(usual answers for BMR: 20–40 kcal/m^2^/h [8])

(usual answers for the Milner equation: 2000–6000 kcal/d)

This initial caloric goal is determined by the Milner equation shown above [1,4], utilizing an activity factor of 1.4 in an effort to maintain weight [5,6,7], along with anabolic agents and physical therapy [8] to maximize lean body mass retention and strength retention. Table 1 shows the general initial nutrition recommendations, split by a less than 20% TBSA burn and larger burns. Further evaluation by indirect calorimetry and dual X-ray absorptiometry (DEXA) is performed as possible [9]. Body composition analysis via DEXA is a valuable tool for assessing nutritional adequacy and monitoring changes over time in burn patients. Visceral proteins (prealbumin, transferrin, and retinol binding protein) are not measured, as they are not good indicators of nutrition status [9,10,11,12,13]. Assessing the adequacy of nutritional intake is crucial in determining appropriate intervention strategies. The accurate estimation of energy needs and actual intake is critical to prevent malnutrition and promote wound healing in burn patients. The calorie intake from enteral nutrition, parenteral nutrition, intravenous fluids, and oral intake is monitored from admission to healing. A calorie surplus (over 120% of goal) is avoided, as overfeeding can lead to ventilator dependence due to increased carbon dioxide production. The target enteral nutrition rate should be achieved within 48 h of admission, and 80–120% of the kcal goal should be met every day starting on hospital day 3, along with the achievement of a positive nitrogen balance [2]. Wound healing is the primary goal of this aggressive nutrition support.

Carbohydrates are the primary source of energy, and a higher carbohydrate and lower fat intake was found to lower the incidence of pneumonia and wound infection, decrease hospital LOS per percentage of TBSA burn, lower rates of PaO_2_/FiO_2_ < 200, and shorten the time to wound healing per percentage of TBSA burn in two individual RCTs evaluating a low-fat (≤15%) and high-carbohydrate (≥60%) intake [14,15]. When these two RCTs were evaluated together through a meta-analysis, decreased pneumonia rates were found [16].

Adequate fat intake is important for preventing essential fatty acid deficiency; however, only 1–4% of total energy intake as fat is sufficient [17,18]. There are five small RCTs [14,15,19,20,21,22] showing clinical outcome benefits for fish oil, including the following: increased body weight at discharge, less wound infection, less time in the hospital adjusted for burn size, and less severe sepsis and septic shock, though with the drawback of more overall infections when providing fish oil to burn patients.

Protein losses in burn patients are significantly elevated through both urinary losses and wound exudate, with a higher protein uptake in the wound beds due to utilization with wound healing. At least 1.5–2 g protein/kg per day is recommended. Alternatively, 25% of calories from protein can be used as the initial protein goal. Nitrogen balance studies can be conducted to better evaluate losses. Avoiding over 5 gm protein/kg ideal body weight is recommended, even if the nitrogen balance is negative.

The Waxman equation [23] is used to estimate nitrogen losses from open wounds:

nitrogen loss/day over the 1st week (gm) = 0.3 × BSA × %TBSA Open Wound

nitrogen loss/day after the 1st week (gm) = 0.1 × BSA × %TBSA Open Wound

%TBSA Open Wound = total body surface area unhealed burn wound plus total body surface area unhealed donor sites (%) × 100, Ex: 20% TBSA unhealed burn + 20% TBSA unhealed donor, enter “40”

Note: %TBSA Open Wound does change with healing

BSA (m^2^) (usual answers: 1.5–2.5) = body surface area

square root of (HT × WT)/3600

HT = height (cm)

WT = weight (kg) (for obese pts, use actual dry weight)

Nitrogen balance = (gm protein intake/6.25) − [UUN × 1.2 + 2 + Waxman + (ΔBUN)]

ΔBUN = change in blood urea nitrogen during UUN collection

UUN = urine urea nitrogen

Note: UUN inaccurate with renal failure/elevated BUN

## 3. Micronutrients

Burn patients utilize additional vitamin C due to increased oxidative stress and tissue damage [24]. Vitamin C supplementation was shown to enhance collagen synthesis, aid in wound healing, and improve immune function in burn patients [25]. We provide 500 mg/d of vitamin C three times daily for patients with severe burns.

One study found lower levels of vitamin E in patients who died after severe burns compared to survivors [26]. Low vitamin E levels may stem from low vitamin C levels. In animal models, vitamin E supplementation resulted in improved wound healing [27,28,29,30,31]. We provide 400 IU/d of enteral vitamin E daily.

Zinc deficiency is common in burn patients and can impair wound healing and immune function. Zinc supplementation was associated with improved wound healing rates, reduced infection rates, and enhanced immune response in burn patients. Copper deficiency can occur with daily doses of 40 mg elemental zinc [25]. We supplement 50 mg/d of enteral elemental zinc (220 mg of zinc sulfate) for patients with severe burns.

An RCT by Barbosa et al. evaluated vitamin E, vitamin C, and zinc supplementation [32]. Subjects in the treatment group received 1.5 times the upper limit for vitamin E and vitamin C and twice the recommended dietary allowance for zinc for one week starting on the second day of admission and had a shorter time to wound healing (5 ± 1 vs. 8 ± 1 days, *p* < 0.01).

Vitamin A plays a crucial role in wound healing, immune function, and epithelial cell differentiation. Supplementation with vitamin A can help promote re-epithelialization and reduce the risk of infections in burn patients. Although deficiency can impair collagen synthesis and interfere with wound healing, whole-body vitamin A deficiency is rare, as the liver has stores that can last several months [25]. Vitamin A is thought to counteract the effects of corticosteroids and impaired wound healing, based on rat studies [25,33]. Vitamin A toxicity was reported in burn patients not receiving additional supplementation in addition to a multivitamin [34] and can become elevated with acute kidney injury. We do not routinely supplement vitamin A.

Selenium also has antioxidant properties. Burn injuries result in decreased selenium levels, and supplementation may have a beneficial effect on wound healing and infection rates in burn patients [25,35,36]. We supplement 400 mcg/d of enteral selenium daily.

Thiamine supplementation can aid in decreased lactate levels in burn patients [37]. We supplement 100 mg of thiamine daily.

Low vitamin D levels are common for patients with severe burns [38]. Deficiency in the initial post-burn period is as a result of hemodilution with burn resuscitation, and the decrease in carrier proteins such as the vitamin D binding protein and albumin associated with inflammation. Vitamin D deficiency is associated with lower bone mineral density and increased prevalence of long bone fractures [39] as well as with low scar elasticity and decreased skin barrier function [40]. We provide aggressive supplementation of 1250 mg of vitamin D_3_ every 3 days and monitor 25-OH vitamin D levels for adjustments.

Copper deficiency, which is common after severe burns, results in poor wound healing, anemia, and neutropenia [25,41]. We check copper levels every 2 weeks and supplement with 4 mg of IV copper daily for 1–2 weeks when severe deficiencies are identified. For slight decreases in copper levels, enteral zinc supplementation should be discontinued.

If the mean corpuscular volume is high, the vitamin B12, folic acid, methylmolonic acid, and homocysteine levels are evaluated, as macrocytic anemia can be a sign of vitamin B12 or folate deficiency. Elevations in methylmolonic acid and homocysteine are better indicators of vitamin B12 deficiency than vitamin B12 levels. Vitamin B12 is supplemented if a deficiency is found. Folate levels are decreased after a severe burn [42,43]. We supplement 1 mg of folate daily.

If the mean corpuscular volume is low, an iron panel is ordered, as a microcytic anemia can be a sign of iron deficiency. Iron plays a role in wound healing as a cofactor in collagen synthesis but has not been thoroughly studied in burn patients [44]. Iron levels are commonly low after severe burns, with a corresponding elevation in ferritin levels [24,44]. We do not usually supplement iron, as burn patients typically receive many blood transfusions, which can result in iron overload.

If a patient is malnourished, extreme electrolyte disturbances are expected. However, phosphorus also significantly drops in well-nourished, hypermetabolic patients during nutrition initiation and must also be closely monitored and aggressively repleted. Due to this, intravenous sodium phosphate is started at 30 mmol every 6 h with the initiation of enteral nutrition. This practice was reported to result in decreased cardiac events and infections [45]. When unable to obtain phosphorus over 2 mg/dL, potassium over 3 mmol/L, and magnesium over 1 mg/dL, enteral nutrition is held or temporarily decreased until these levels are under control. Individual patient assessments should guide the need for specific micronutrient supplementation.

## 4. Enteral Nutrition

Enteral nutrition (EN) is the preferred route of nutrition, as it aids in maintaining gut integrity, preserving immune function, and reducing the risk of infection. The initial goal for a burn patient is to start enteral nutrition as soon as clinically appropriate, within the first 24 h of admission [9,46,47,48,49,50,51,52,53,54,55,56]. One RCT evaluating the timing of EN initiation found a significant difference in body mass index changes with early EN (ranging from 0 to 24 h after admission) in burn patients [55]. A multicenter, observational study found lower rates of wound infection and a shorter intensive care unit length of stay to be associated with initiating EN within 24 h of admission [57].

We use an enteral formulation with high protein and high carbohydrate provisions. Diabetic, renal, pulmonary, acute respiratory distress syndrome, and concentrated enteral nutrition formulas are not routinely used, as these are all high-fat. Low-fat and high-carbohydrate formulas were found to improve healing and other outcomes in burn patients [14,15,16,58,59,60]. Immune-enhancing formulas are not utilized, as the high arginine levels increase mortality in patients with sepsis. For diabetes, an insulin drip is started as needed (see ISR 1-120 BICU Insulin Clinical Practice Guidelines). For renal failure, continuous renal replacement therapy is utilized when needed.

Since the guidelines [9] recommend waiting to start enteral nutrition until hemodynamic stability is achieved, albeit with no definition of hemodynamic stability, we clinically defined hemodynamic stability as lactate beginning to normalize (<3 mmol/L) and epinephrine or norepinephrine infusions under 0.15 mcg/kg/min and await these criteria before initiating enteral nutrition. Bowel sounds, stool output, and flatus are not used to determine if enteral nutrition can be initiated, as they are not good signs of bowel function [9]. Enteral nutrition is initiated at 20 mL/h and increased by 20 mL/h every 4 h as tolerated, until the goal rate is achieved. Boluses of 5–6 g of protein powder are scheduled every three hours with the initiation of enteral nutrition, and these boluses are increased by 5–6 g each day until the goal is reached. Dosing can later be adjusted to maintain a positive nitrogen balance (equation shown above) but cannot be decreased below the initial estimate until ~hospital day 14, as urine urea nitrogen levels trend up during this time. When the initial EN goal is achieved, and the phosphorus level is able to be maintained above 3 mg/dL, a maltodextrin mixture (of approximately 2 kcal/mL) is added to the flush bag, and enteral nutrition is adjusted to provide 60–65% of carbohydrates and at least 25% of the kcal from protein (or per the nitrogen balance results if higher). A minimum of a 30 mL water flush every four hours is required to keep feeding tube patent. Intravenous fluids are minimized as tolerated as enteral nutrition reaches the goal.

The enteral nutrition hourly rate can be increased by approximately 20–40 mL/h the day prior to surgery and decreased to the original rate the day after to account for the time in surgery. Post-pyloric enteral nutrition can be held at the time of transport to surgery in patients with protected airways. Otherwise, enteral nutrition formulas can be held six hours prior to surgery. Clear liquids (e.g., clear supplement drinks or a maltodextrin mixture) can be continued orally, in the stomach, or post-pyloric until two hours before surgery,. Toast or cereal (not with milk) can be allowed up to six hours before surgery. When there is a nasogastric/orogastric tube, the stomach is suctioned out in both the intensive care unit room prior to leaving for surgery and in the operating room prior to starting surgery to aid in preventing aspiration. Enteral nutrition can be resumed at the pre-surgical rate when the patient is hemodynamically stable after surgery [61]. Enteral nutrition is not held for vasopressin or dobutamine. Enteral nutrition is held for epinephrine or norepinephrine at 0.15 mcg/kg/min. A catch-up rate is ordered to allow the nursing staff to automatically account for any time enteral nutrition is missed: the current enteral nutrition goal rate is increased by one-third for 3 h for every one hour of enteral nutrition missed [61].

## 5. Enteral Nutrition in the Austere Environment

In past conflicts such as Operation Iraqi Freedom and Operation Enduring Freedom, the United States maintained control of the airspace and, with that, casualties were evacuated out of the theater and to a level 5 facility in the United States within 2–3 days of the point of injury [62,63]. Due to this, the average length of stay at a combat support hospital (CSH) was only about 17.4 h, which includes any required surgery and preparation for evacuation, leaving little time for a nutrition evaluation by a dietitian [63]. As a result, there are limited published data on providing nutrition support in an austere environment or warzone.

It is impractical, both logistically and economically, to produce, transport, and store prepared commercial enteral nutrition formulas in developing nations and austere environments [64]. Not only are there limitations on storing enteral products, but the transportation of such products can be dangerous and is often trumped by higher priority items such as water and munitions [64].

In an article by Frizzi et al. [64], surgeons on a forward surgical team (FST) in Afghanistan were presented with the challenge of providing early enteral nutrition to seven injured service members and Afghan nationals awaiting combat zone evacuation. All suffered major trauma, but the authors were unclear about the mechanism of injuries to include if any of these individuals suffered from burns. The FST employed the use of nasogastric tubes for patients not requiring diagnostic or therapeutic laparotomies and surgical gastrostomy/jejunostomy tubes for those undergoing a laparotomy. After the first 12–24 h post op where the gastrostomy tube was on gravity draining, enteral feeds were started with 1% low-fat milk at a rate of 30 mL/h. The preferred method of feeding was through bolus feeds, as the FSTs were not equipped with feeding pumps, but if a continuous rate was needed, a 100 mL intravenous bag of saline was substituted as the tube feed bag and a dial-a-flow was placed to control the rate of infusion. The enteral rate was increased to 60 mL/h following tolerance to trickle feeds; additionally, once tolerance was established, the Meal, Ready-to-Eat (MRE) dairy shake powder was added to the milk feeding to provide additional calories and nutrients. Small batches of feeds were prepared to prevent the separation of additives, and feeding rates were decreased as the oral intake increased.

Homemade enteral formulas are a viable option when it comes to austere environments; however, consideration needs to be taken as to how to provide the required nutrition despite the constraints of the situation and local environment. Food stuffs may need to be procured via the local economy, and, depending on the region, dietary and cultural restrictions may further dictate the available food products [64]. Stankorb et al. discussed a rudimentary concoction made from milk, honey, and eggs due to lack of available commercially prepared options that was used for tube feeding by an Iraqi hospital [63]. Time and temperature controls also need to be considered for both the storing of ingredients and the holding of prepared mixtures. The addition of a blender, food processor, or hand-cranked food mill with a clean and reliable supply of water and electricity is required for the preparation of enteral feeds [64].

## 6. Parenteral Nutrition

Commercial parenteral formulas are not available far forward (i.e., at the FST) on the battlefield [64]. The appropriate initiation of parenteral nutrition is an area of evolving evidence, and clinical practice is only indicated when enteral nutrition is not feasible because of compromised gastrointestinal function or when enteral nutrition fails to meet the patient’s nutritional needs. The following guidelines are based on the available literature. Consider parenteral nutrition for high-mortality-risk pts. per the Baux score (e.g., ≥60% predicted mortality, especially in those patients outside a body mass index of 25–35 kg/m^2^) if unable to achieve the enteral nutrition goal rate within two days [65,66]. Consider parenteral nutrition for moderate-mortality-risk patients if unable to achieve the enteral nutrition goal rate within five days. Consider parenteral nutrition for low-mortality-risk patients with a good nutrition status prior to injury if unable to achieve the enteral nutrition goal rate after seven days [9].

Start with an initial dextrose infusion rate (see below) of five. This can be increased to seven if well tolerated (low/no insulin requirements). We provide intravenous amino acids at the estimated protein goal (25% of kcals or based off the nitrogen balance if available) with the remainder of calories coming from dextrose, up to a dextrose infusion rate of seven.

$$\mathrm{Dextrose}\;\mathrm{infusion}\;\mathrm{rate}=\frac{calories\;from\;dextrose\times 1000}{3.4\times weight(kg)\times 1440}$$

We give 15% of the caloric goal in the form of SMOF (soybean, medium-chain triglycerides, olive oil, and fish oil) lipids. Prior to the availability of SMOF lipids, we did not usually give any intravenous lipids during parenteral nutrition unless there was no fat intake for seven days, including fat from enteral nutrition and propofol [10]. When needed, 500 calories from intravenous lipids were given twice weekly to prevent essential fatty acid deficiency. Triglycerides are monitored at least weekly when receiving any IV lipids.

Direct bilirubin and liver function panels are monitored each week while on parenteral nutrition. Copper and manganese are held if the direct bilirubin is 2 mg/dL or greater.

Efforts to achieve the enteral nutrition goal rate are actively continued even after parenteral nutrition is initiated in order to discontinue parenteral nutrition as soon as possible. Trophic enteral nutrition can be started with a suspected ileus and may help the ileus to resolve [10]. To wean off parenteral nutrition, the rate can be decreased by the tolerated enteral nutrition rate.

## 7. Oral Nutrition

A regular, non-restricted diet is given if no intubation occurs. Otherwise, the diet is advanced when the patient is extubated (and no trach) or when the trach is downsized to #6, off the ventilator. An appropriate mental status is ensured prior to oral intake, with clear speech and the ability to manage secretions. If the patient has both a Dobb-Hoff tube and a nasogastric/orogastric tube, one tube is removed to allow for ease of swallowing. A pudding texture is initially given during the bedside nursing swallowing evaluation, and the diet is advanced as tolerated to a regular diet with only supplements and milk for fluids. A clear liquid diet → full liquid diet → soft diet → regular diet pattern of advancement is not appropriate, as this has no scientific basis; it prolongs diet advancement and contributes to inadequate nutrition. To aid in the transition from enteral to oral nutrition, enteral nutrition is held for two hours for each supplement the patient drinks or meal the patient eats. Do not use diet restrictions, such as diabetic, renal, heart healthy, etc., until the patient is tolerating an adequate amount of oral intake to warrant these; the patient should be able to decide what foods they will eat, as they do at home. If inappropriate diet choices are made, this is an opportunity for re-education: poor diet choices will not be identified if foods are limited by the diet order. Calorie counts are performed to ensure the patient is meeting nutrition goals. No water, Gatorade, Kool-Aid, or caffeine-containing beverages are allowed, as these are not calorically dense enough to support the hypermetabolic state and water intoxication (severe hyponatremia) that can develop if patients with the overactive thirst mechanism associated with a burn are allowed to drink all of the desired fluids. Only supplements and milk are initially given for fluids until the patient is meeting their calorie goal and experiencing no difficulty with hyponatremia. Typically, burn patients are not hungry [67] but may be extremely thirsty, which aids them in drinking a large number of supplement drinks. The dietitian provides a supplement goal (usual goal: 8–24 supplements per day).

## 8. Medications for the Hypermetabolic Response

Insulin is the best anabolic steroid and is used as a tool to aid in achieving the estimated nutrition needs [68]. Possible adverse outcomes include hypoglycemia. We initiate an insulin drip when glucose is over 180 mg/dL twice in a row. Carbohydrate provisions are not altered unless the insulin drip rate reaches 75 units/h in our facility, and then the enteral nutrition rate is temporarily decreased, as this high insulin requirement is usually temporary. The enteral nutrition rate is returned to goal if able to keep the insulin drip under 75 units/h. Long-acting insulin is avoided in patients with normal hemoglobin A1c values on admission, as long-acting insulin may cause difficulty with hypoglycemia when the inflammatory state resolves. The goal glucose levels are between 80 and 180 mg/dL.

Oxandrolone, a testosterone analogue, was found to increase lean body mass retention and decrease the length of hospital stay [69]. Possible adverse outcomes include alanine transaminase >100 U/L. We start oxandrolone on post-burn day five at a dose of 10 mg twice per day. Liver function tests are monitored weekly. Oxandrolone is held for alanine transaminase >200 U/L and (re)started when <100 U/L. The goal is lean body mass retention.

Propranolol was found to decrease metabolism and increase lean body mass retention (in burned children). Possible adverse outcomes include bradycardia and hypotension. The hold parameters for heart rate and/or blood pressure should be included in the administration plan [70]. We start propranolol on approximately post-burn day 5 with a dose of 10 mg given enterally twice per day/three times per day. This dosing is increased every other day by 5 mg per dose with the goal of a 20% decrease in the heart rate, as this was found to decrease the hypermetabolic response.

## 9. Conclusions

Nutrition plays a critical role in the recovery and wound healing of burn patients. Adequate carbohydrate, protein, and micronutrient intake is an essential treatment for hypermetabolic and hypercatabolic responses. A high value should be placed on ensuring adequate nutrition is provided to a burn patient to promote recovery.

The current information available on nutrition support in the austere environment is extremely limited. The management of patient feeding for the different roles of care in the deployed setting needs to be shared with the nutrition community at large for improvements in the clinical outcomes in these environments to take place.

## Figures and Tables

**Table 1:** General initial macronutrient and micronutrient recommendations.

	≥20% TBSA Burn	<20% TBSA Burn
Energy	Milner equation	35 kcal/kg
Protein	~25% energy goal initially, and then +nitrogen balance	~20% energy goal initially and then +nitrogen balance
Carbohydrate	60–65% energy goal	
Fat	~10–15% energy goal	
Vitamin C	500 mg of TID	500 mg daily
Vitamin E	400 IU daily	
Zinc	50 mg of elemental/ 220 mg zinc sulfate daily	
Selenium	400 mcg daily	
Thiamine	100 mg daily	
Folate	1 mg daily	
Vitamin D_3_	20,000 IU Q3D	
Phosphorus	30 mmol of IV sodium phosphate Q6H	
Multivitamin with minerals	1 tab daily	1 tab daily

TBSA = total body surface area; TID = three times daily; Q3D = every three days; Q6H = every 6 h.

## References

[1] Milner E.A., Cioffi W.G., Mason A.D., McManus W.F., Pruitt B.A. (1994). A longitudinal study of resting energy expenditure in thermally injured patients. J. Trauma..

[2] American Society for Parenteral and Enteral Nutrition (2007). Nutrition Support Core Curriculum: A Case Based Approach to the Adult Patient.

[3] Saffle J.R., Larson C.M., Sullivan J. (1990). A randomized trial of indirect calorimetry-based feedings in thermal injury. J. Trauma..

[4] Shields B.A., Doty K.A., Chung K.K., Wade C.E., Aden J.K., Wolf S.E. (2013). Determination of resting energy expenditure after severe burn. J. Burn. Care Res..

[5] Hart O.W., Wolf S.E., Herndon O.N., Chinkes D.L., Lal S.O., Obeng M.K., Beauford R.B., Mlcak R.P. (2002). Energy expenditure and caloric balance after burn: Increased feeding leads to fat rather than lean mass accretion. Ann. Surg..

[6] Goran M.I., Peters E.J., Herndon O.N., Wolfe R.R. (1990). Total energy expenditure in burned children using the doubly labeled water technique. Am. J. Physiol..

[7] Garrel D.R., de Jonge L. (1993). Thermogenic response to feeding in severely burned patients: Relation to resting metabolic rate. Burns.

[8] Shields B.A., Carpenter J.N., Bustillos B.D., Jordan A.N., Cunningham K.B., Vegas S.J., Aden J.K., Rowan M.P., Rizzo J.A., Dewey W.S. (2019). The interplay of nutrition, physical activity, severity of illness, and mortality in critically ill burn patients: Is there a connection?. J. Burn. Care Res..

[9] Martindale R.G., McClave S.A., Vanek V.W., McCarthy M., Roberts P., Taylor B., Ochoa J.B., Napolitano L., Cresci G., American College of Critical Care Medicine (2009). Guidelines for the provision and assessment of nutrition support therapy in the adult critically ill patient: Society of Critical Care Medicine and American Society for Parenteral and Enteral Nutrition: Executive summary. Crit. Care Med..

[10] Gabay C., Kushner I. (1999). Acute-phase proteins and other systemic responses to inflammation. N. Engl. J. Med..

[11] Fuhrman M.P., Charney P., Mueller C.M. (2004). Hepatic proteins and nutrition assessment. J. Am. Diet. Assoc..

[12] Shields B.A., Pidcoke H.F., Chung K.K., Wade C.E., Martini W.Z., Renz E.M., Wolf S.E. (2015). Are visceral proteins valid markers for nutritional status in hyper calorically fed critically ill burn patients?. J. Burn. Care Res..

[13] Carlson D.E., Cioffi W.G., Mason A.D., McManus W.F., Pruitt B.A. (1991). Evaluation of serum visceral protein levels as indicators of nitrogen balance in thermally injured patients. J. Parenter. Enteral Nutr..

[14] Gottschlich M.M., Jenkins M., Warden G.D., Baumer T., Havens P., Snook J.T., Alexander J.W. (1990). Differential effects of three enteral dietary regimens on selected outcome variables in burn patients. J. Parenter. Enteral Nutr..

[15] Garrel D.R., Razi M., Larivière F., Jobin N., Naman N., Emptoz-Bonneton A., Pugeat M.M. (1995). Improved clinical status and length of care with low-fat nutrition support in burn patients. J. Parenter. Enteral Nutr..

[16] Masters B., Aarabi S., Sidhwa F., Wood F. (2012). High-carbohydrate, high-protein, low-fat versus low-carbohydrate, high-protein, high-fat enteral feeds for burns. Cochrane Database Syst. Rev..

[17] Wiese H.F., Hansen A.E., Adam D.J. (1958). Essential fatty acids in infant nutrition. I. Linoleic acid requirement in terms of serum di-, tri- and tetraenoic acid levels. J. Nutr..

[18] Bistrian B.R. (2003). Clinical aspects of essential fatty acid metabolism: Jonathan Rhoads Lecture. J. Parenter. Enter. Nutr..

[19] Saffle J.R., Wiebke G., Jennings K., Morris S.E., Barton R.G. (1997). Randomized trial of immune-enhancing enteral nutrition in burn patients. J. Trauma..

[20] Wibbenmeyer L.A., Mitchell M.A., Newel I.M., Faucher L.D., Amelon M.J., Ruffin T.O., Lewis R.D., Latenser B.A., Kealey P.G. (2006). Effect of a fish oil and arginine-fortified diet in thermally injured patients. J. Burn. Care Res..

[21] Iamsirisaengthong W., Chinaroonchai K., Chuntrasakul C., Roeksomtawin S., Muangman P. (2017). Prospective controlled trial to compare immune-enhancing and regular enteral diets to reduce septic complication in major burn patients. J. Med. Assoc. Thail..

[22] Tihista S., Echavarría E. (2018). Effect of omega 3 polyunsaturated fatty acids derived from fish oil in major burn patients: A prospective randomized controlled pilot trial. Clin. Nutr..

[23] Waxman K., Rebello T., Pinderski L., O’Neal K., Khan N., Tourangeau S., Himes E., Cordill K. (1987). Protein loss across burn wounds. J. Trauma..

[24] Vinha P.P., Martinez E.Z., Vannucchi H., Marchini J.S., Farina J.A., Jordao A.A., Cunha S.F. (2013). Effect of acute thermal injury in status of serum vitamins, inflammatory markers, and oxidative stress markers: Preliminary data. J. Burn. Care Res..

[25] McKeever L., Mueller C.M. (2017). Vitamins and Trace Elements. The ASPEN Adult Nutrition Support Core Curriculum eBook.

[26] Nguyen T.T., Cox C.S., Traber D.L., Gasser H., Redl H., Schlag G., Herndon D.N. (1993). Free radical activity and loss of plasma antioxidants, vitamin E, and sulfhydryl groups in patients with burns: The 1993 Moyer Award. J. Burn. Care Rehabil..

[27] Houwing R., Overgoor M., Kon M., Jansen G., van Asbeck B.S., Haalboom J.R. (2000). Pressure-induced skin lesions in pigs: Reperfusion injury and the effects of vitamin E. J. Wound Care.

[28] Musalmah M., Fairuz A.H., Gapor M.T., Ngah W.Z. (2002). Effect of vitamin E on plasma malondialdehyde, antioxidant enzyme levels and the rates of wound closures during wound healing in normal and diabetic rats. Asia Pac. J. Clin. Nutr..

[29] Taren D.L., Chvapil M., Weber C.W. (1987). Increasing the breaking strength of wounds exposed to preoperative irradiation using vitamin E supplementation. Int. J. Vitam. Nutr. Res..

[30] Ehrlich H.P., Tarver H., Hunt T.K. (1972). Inhibitory effects of vitamin E on collagen synthesis and wound repair. Ann. Surg..

[31] Morita N., Shimoda K., Traber M.G., Westphal M., Enkhbaatar P., Murakami K., Leonard S.W., Traber L.D., Traber D.L. (2006). Vitamin E attenuates acute lung injury in sheep with burn and smoke inhalation injury. Redox Rep..

[32] Barbosa E., Faintuch J., Machado Moreira E.A., da Silva V.R.G., Pereima M.J.L., Fagundes R.L.M., Filho D.W. (2009). Supplementation of vitamin E, vitamin C, and zinc attenuates oxidative stress in burned children: A randomized, double-blind, placebo-controlled pilot study. J. Burn. Care Res..

[33] Wicke C., Halliday B., Allen D., Roche N.S., Scheuenstuhl H., Spencer M.M., Roberts A.B., Hunt T.K. (2000). Effects of steroids and retinoids on wound healing. Arch. Surg..

[34] Bremner N.A., Mills L.A., Durrani A.J., Watson J.D. (2007). Vitamin A toxicity in burns patients on long-term enteral feed. Burns.

[35] Berger M.M., Cavadini C., Bart A., Blondel A., Bartholdi I., Vandervale A., Krupp S., Chiolero R., Freeman J., Dirren H. (1992). Selenium losses in 10 burned patients. Clin. Nutr..

[36] Dylewski M.L., Bender J.C., Smith A.M., Prelack K., Lydon M., Weber J.M., Sheridan R.L. (2010). The selenium status of pediatric patients with burn injuries. J. Trauma..

[37] Falder S., Silla R., Phillips M., Rea S., Gurfinkel R., Baur E., Bartley A., Wood F.M., Fear M.W. (2010). Thiamine supplementation increases serum thiamine and reduces pyruvate and lactate levels in burn patients. Burns.

[38] Gottschlich M.M., Mayes T., Khoury J., Kagan R.J. (2017). Clinical trial of vitamin D_2_ vs D_3_ supplementation in critically ill pediatric burn patients. J. Parenter. Enter. Nutr..

[39] Krishnan A., Ochola J., Mundy J., Jones M., Kruger P., Duncan E., Venkatesh B. (2010). Acute fluid shifts influence the assessment of serum vitamin D status in critically ill patients. Crit. Care.

[40] Cho Y.S., Seo C.H., Joo S.Y., Song J., Cha E., Ohn S.H. (2019). The association between postburn vitamin D deficiency and the biomechanical properties of hypertrophic scars. JBCR.

[41] Voruganti V.S., Klein G.L., Lu H.X., Thomas S., Freeland-Graves J.H., Herndon D.N. (2005). Impaired zinc and copper status in children with burn injuries: Need to reassess nutritional requirements. Burns.

[42] Barlow G.B., Wilkinson A.W. (1970). 4-amino-imidazole-5-carboxamide excretion and folate status in children with burns and scalds. Clin. Chim. Acta.

[43] Zhang X.J., Chinkes D.L., Herndon D.N. (2008). Folate stimulation of wound DNA synthesis. J. Surg. Res..

[44] Żwierełło W., Styburski D., Maruszewska A., Piorun K., Skórka-Majewicz M., Czerwińska M., Maciejewska D., Baranowska-Bosiacka I., Krajewski A., Gutowska I. (2020). Bioelements in the treatment of burn injuries—The complex review of metabolism and supplementation (copper, selenium, zinc, iron, manganese, chromium and magnesium). J. Trace Elem. Med. Biol..

[45] Kahn S.A., Bell D.E., Stassen N.A., Lentz C.W. (2015). Prevention of hypophosphatemia after burn injury with a protocol for continuous, preemeptive repletion. J. Burn. Care Res..

[46] Chiarelli A., Enzi G., Casadei A., Baggio B., Valerio A., Mazzoleni F. (1990). Very early nutrition supplementation in burned patients. Am. J. Clin. Nutr..

[47] Enzi G., Casadei A., Sergi G., Chiarelli A., Zurlo F., Mazzoleni F. (2006). Metabolic and hormonal effects of early nutritional supplementation after surgery in burn patients. Crit. Care Med..

[48] Scott McDonald W., Sharp C.W., Deitch E.A. (2006). Immediate enteral feeding in burn patients is safe and effective. Ann. Surg..

[49] Engelbrecht V.J., Clarke S.M. (1994). Early enteral feeding of a severely burned pediatric patient. J. Burn. Care Rehabil..

[50] Klasen H.J., ten Duis H.J. (1987). Early oral feeding of patients with extensive burns. Burns.

[51] Garrel D.R., Davignon I., Lopez D. (1991). Length of care in patients with severe burns with or without early enteral nutritional support. J. Burn. Care Rehabil..

[52] Jenkins M., Gottschlich M., Alexander J.W. (1994). An evaluation of the effect of immediate enteral feeding on the hypermetabolic response following severe burn injury. J. Burn. Care Rehabil..

[53] Gottschlich M.M., Jenkins M.E., Mayes T., Khoury J., Kagan R.J., Warden G.D. (2002). An evaluation of the safety of early vs delayed enteral support and effects on clinical, nutritional, and endocrine outcomes after severe burns. J. Burn. Care Rehabil..

[54] Peck M.D., Kessler M., Cairns B.A., Chang Y.-H., Ivanova A., Schooler W. (2004). Early enteral nutrition does not decrease hypermetabolism associated with burn injury. J. Trauma..

[55] Vicic V.K., Radman M., Kovacic V. (2013). Early initiation of enteral nutrition improves outcomes in burn disease. Asia Pac. J. Clin. Nutr..

[56] Ostadrahimi A., Nagili B., Asghari-Jafarabadi M., Beigzali S., Zalouli H., Lak S. (2016). A proper enteral nutrition support improves sequential organ failure score and decreases length of stay in hospital in burned patients. Iran. Red. Crescent Med. J..

[57] Mosier M.J., Pham T.N., Klein M.B., Gibran N.S., Arnoldo B.D., Gamelli R.L., Tompkins R.G., Herndon D.N. (2011). Early enteral nutrition in burns: Compliance with guidelines and associated outcomes in a multicenter study. JBCR.

[58] Cree M.G., Aarsland A., Herndon D.N., Wolfe R.R. (2007). Role of fat metabolism in burn trauma-induced skeletal muscle insulin resistance. Crit. Care Med..

[59] Hart D.W., Wolf S.E., Zhang X.-J., Chinkes D.L., Buffalo M.C., Matin S.I., DebRoy M.A., Wolfe R.R., Herndon D.N. (2001). Efficacy of a high-carbohydrate diet in catabolic illness. Crit. Care Med..

[60] Lee J.O., Gauglitz G.G., Herndon D.N., Hawkins H.K., Halder S.C., Jeschke M.G. (2011). Association between dietary fat content and outcomes in pediatric burn patients. J. Surg. Res..

[61] Shields B.A., Brown J.N., Aden J.K., Salgueiro M., Mann-Salinas E.A., Chung K.K. (2014). A pilot review of gradual versus goal re-initiation of enteral nutrition after burn surgery in the hemodynamically stable patient. Burns.

[62] Stankorb S.M., Salgueiro M., Grediagin A. (2009). Enteral Feeding Practices for U.S. Service Members in a Deployed Combat Support Hospital. Mil. Med..

[63] Stankorb S.M., Ramsey C., Clark H., Osgood T. (2014). Provision of Nutrition Support Therapies in the Recent Iraq and Afghanistan Conflicts. Nutr. Clin. Pract..

[64] Frizzi J.D., Ray P.D., Raff J.B. (2005). Enteral Nutrition by a Forward Surgical Team in Afghanistan. South. Med. J..

[65] Wischmeyer P. (2012). Parenteral nutrition and calorie delivery in the ICU: Controversy, clarity, or call to action?. Curr. Opin. Grit Care.

[66] Canadian Clinical Practice Guidelines May 2015, Canadian Clinical Practice Guidelines: Strategies to Optimize Parenteral Nutrition and Minimize Risks: Use of Lipids. https://criticalcarenutrition.com/docs/CPGs%202015/10.2%202015.pdf.

[67] Wade C.E., Mora A.G., Shields B.A., Pidcoke H.F., Baer L.A., Chung K.K., Wolf S.E. (2013). Signals from fat after injury: Plasma adipokines and ghrelin concentrations in the severely burned. Cytokine.

[68] Aarsland A., Chinkes D.L., Sakurai Y., Nguyen T.T., Herndon D.N., Wolfe R.R. (1998). Insulin therapy in burn patients does not contribute to hepatic triglyceride production. J. Clin. Investig..

[69] Wolf S.E., Edelman L.S., Kemalyan N., Donison L., Cross J., Underwood M., Spence R.J., Noppenberger D., Palmieri T.L., Greenhalgh D.G. (2006). Effects of oxandrolone on outcomes measures in the severely burned: A multicenter prospective randomized double-blind trial. J. Burn. Care Res..

[70] Herndon D.N., Hart D.W., Wolf S.E., Chinkes D.L., Wolfe R.R. (2001). Reversal of catabolism by beta-blockade after severe burns. NEJM.

